# Supplementary material for: Structure of the active Gi-coupled human lysophosphatidic acid receptor 1 complexed with a potent agonist
Source: Nat Commun. 2022 Sep 15;13:5417. doi: 10.1038/s41467-022-33121-2 (PMC9477835; doi:10.1038/s41467-022-33121-2)
Supplement: Supplementary file 1 — Supplementary Information [file 41467_2022_33121_MOESM1_ESM.pdf]

Supplementary Information

Structure of the active G_i-coupled human Lysophosphatidic Acid Receptor 1 complexed with a potent agonist

Hiroaki Akasaka, Tatsuki Tanaka, Fumiya K. Sano, Yuma Matsuzaki, Wataru Shihoya, Osamu Nureki.

	pEC$_{50}$		E_{max}	
	Mean ± S.E.M.	EC$_{50}$ (nM)	Mean ± S.E.M.	*n*
LPA	8.10 ± 0.06	8.0	0.31 ± 0.01	3
ONO-0740556	9.59 ± 0.01	0.26	0.32 ± 0.01	3

Supplementary Table 1 | Pharmacological characterization of ONO-0740556.
Ligand-induced G_i activation by LPA_1. G_i activation was measured by the NanoBiT-G-protein dissociation assay.
Means ± s.e.m. from three independent experiments was shown.

	LPA1-Gi complex (stable state)	Receptor	State 1	State 2	State 3	State 4
PBD	7YU3	7YU4	7YU5	7YU6	7YU7	7YU8
EMDB	EMD-34097	EMD-34098	EMD-34099	EMD-34100	EMD-34101	EMD-34102
**Data collection**						
Microscope	Titan Krios (Thermo Fisher Scientific)					
Voltage (keV)	300					
Detector	Gatan K3 summit camera (Gatan)					
Magnification	x 105,000					
Electron dose (e-/Å^2)	50					
Defocus range (μm)	-0.8－-1.6					
Pixel size (Å/pix)	0.83					
Number of movies	6,227					
Symmetry	C1					
Picked particles	3,021,676					
Final particles	181.071		129,275	127,465	55,709	51,335
Map resolution (Å)	3.5	3.7	3.7	3.9	4.5	5.6
FSC threshold	0.143					
**Model refinement**						
Atoms	8,788	2,050	8,788	8,788	8,770	8,756
R.M.S.Deviations from ideal						
Bond length	0.0138	0.0124	0.0146	0.0148	0.0145	0.0155
Bond angle	1.51	1.37	1.70	1.76	1.68	1.87
**Validation**						
Clashscore	9.65	9.42	10.17	10.57	10.89	13.47
Rotamer outliers (%)	0.52	0	0.75	0.86	1.29	1.30
Ramachandran plot						
Preferred (%)	95.18	96.12	95.27	95.36	94.91	95.00
Allowed (%)	4.73	3.83	4.55	4.28	4.91	4.91
Outlier (%)	0.09	0	0.18	0.36	0.18	0.09

Supplementary Table 2 | Cryo-EM data collection, refinement and validation statistics

	pEC_{50}		E_{max}		Expression	
	Mean ± S.E.M.	EC_{50} (nM)	Mean ± S.E.M.	*n*	Mean ± S.E.M.	*n*
WT	9.38 ± 0.03	0.42	0.26 ± 0.002	3	100.0	4
WT(1:5)	9.44 ± 0.11	0.36	0.23 ± 0.01	3	56.5 ± 3.4	4
Y34$^{N\text{-}term}$A	8.94 ± 0.05	1.14	0.40 ± 0.01	3	47.3 ± 1.1	3
K39$^{N\text{-}term}$A	7.20 ± 0.08	63.4	0.34 ± 0.01	3	53.9 ± 7.7	4
R124$^{3.28}$A	7.39 ± 0.06	40.7	0.43 ± 0.03	3	111.0 ± 10.7	4
W210$^{5.43}$A	N/A	N/A	N/A	3	81.4 ± 9.0	4
L278$^{6.55}$A	8.81 ± 0.03	1.6	0.26 ± 0.02	3	111.6 ± 13.8	3
L297$^{7.39}$A	8.10 ± 0.06	8.4	0.17 ± 0.02	3	74.0 ± 9.5	4

Supplementary Table 3 | Mutant experiments
NanoBiT-G-protein dissociation assays for LPA1 and its mutants. Means ± s.e.m. from three independent experiments are shown.
Cell surface expression levels assessed by ELISA (supplementary Fig. 4). Means ± s.e.m. from three or four independent experiments are shown.

	LPA_1	$S1P_1$	$S1P_2$	$S1P_3$	$S1P_4$	$S1P_5$	CB_1	CB_2
N-term	**Y**	**Y**	**Y**	**Y**	**Y**	**Y**	N/A	N/A
N-term	**K**	**K**	E	**K**	R	**K**	N/A	N/A
2.60	L	N	N	N	N	N	K	K
ECL1	T	S	S	S	S	S	N/A	N/A
ECL1	**T**	**T**	**T**	**T**	**T**	**T**	N/A	N/A
3.28	**R**	**R**	**R**	**R**	**R**	**R**	K	K
3.29	Q	E	E	E	E	E	L	I
3.32	I	M	A	M	L	V	V	V
3.33	D	F	F	F	F	F	T	T
ECL2	**Y**	**Y**	**Y**	**Y**	**Y**	**Y**	N/A	N/A
5.40	**L**	I	V	I	I	V	**L**	**L**
5.43	**W**	C	V	C	C	C	**W**	**W**
6.51	**G**	L	A	L	L	L	L	V
6.55	**L**	**L**	**L**	F	**L**	**L**	M	M
7.35	E	A	A	A	M	A	F	F
7.36	K	E	H	Q	D	D	A	A
7.38	**F**	**F**	**F**	**F**	I	**F**	C	C
7.39	**L**	**L**	F	I	**L**	**L**	S	S

Supplementary Table 4 | Conservation of the residues involved in agonist binding
Comparison of the residues involved in ONO-0740556 binding among the lipid-sensing GPCRs.

Supplementary Method

Synthesis of ONO-0740556

NMR spectra were recorded as designated on either a Varian (Agilent) Mercury 300 spectrometer or Agilent VNMRS 600 spectrometer using deuterated chloroform ($CDCl_3$) or deuterated dimethyl sulfoxide (DMSO-d6) as the solvent. LC/MS analysis was carried out by Shimadzu Nexera X2 system: Column; YMC Triart C18 (2.0 mm × 30 mm). Gradient condition; 5% acetonitrile in H_2O containing 0.1%TFA (0-0.10 min), 5-95% (0.10-1.20 min), 95% (1.20-1.50 min). Flow rate of 1.0 mL/min, column temperature of 30 °C, detection with UV (PDA) and ELSD. Column chromatography was carried out on silica gel [Merck Silica Gel 60 (0.063–0.200 μm) or Fuji Silysia FL60D]. Thin layer chromatography was performed on silica gel (Merck TLC or HPTLC plates, Silica Gel 60 F254). The following abbreviations for solvents and reagents are used; 1,1'-bis (diphenylphosphino)ferrocene (dppf), dichloromethane (CH_2Cl_2), ethyl acetate (EtOAc), 1-ethyl-3-(3-dimethylaminopropyl)carbodiimide hydrochloride (EDC·HCl), 1-hydroxybenzotriazole (HOBt),.methanol (MeOH), sodium bis(trimethylsilyl)amide (NaHMDS), tetrahydrofuran (THF), triethylamine (Et_3N) .

2-(1-hexyn-1-yl)benzaldehyde (2)

A mixture of 1-hexyne (949 mg, 11.6 mmol), 2-bromobenzaldehyde (1, 1.4 g, 7.7 mmol), Pd(dppf)Cl_2 (169 mg, 0.23 mmol) and CuI (88 mg, 0.46 mmol) in THF (10 mL) and Et_3N (10 mL) was stirred at 95 °C overnight. The reaction mixture was filtered and the resulting filtrate was concentrated under reduced pressure. The residue was purified by silica gel column chromatography (petroleum ether) to give 2 (1.3 g, 6.8 mmol) in 88% yield. TLC: Rf 0.78 (n-hexane:EtOAc = 4:1); 1H-NMR ($CDCl_3$): δ 0.96 (t, J = 7.3 Hz, 3H), 1.45-1.54 (m, 2H), 1.58-1.68 (m, 2H), 2.49 (t, J = 7.1 Hz, 2H), 7.35-7.41 (m, 1H), 7.48-7.55 (m, 2H), 7.87-7.91 (m, 1H), 10.54 (s, 1H).

5-[2-(1-hexyn-1-yl)phenyl]-4-pentenoic acid (3)

To a suspension of (4-carboxybutyl)(triphenyl)phosphonium bromide (4.4 g, 10.2 mmol) in THF (105 mL) was added NaHMDS (3.7 g, 20.3 mmol) dropwise at 0 °C. The mixture was stirred for 3 hours at room temperature. Then a solution of 2 (1.3 g, 6.8 mmol) in THF (15 mL) was added dropwise. After the reaction mixture was stirred for 3 hours, the reaction was quenched with sat. NH_4Cl solution and acidified with 2N HCl to pH = 5~6. The resulting mixture was extracted with EtOAc and the organic layer was washed with brine. The organic layer was dried with anhydrous $MgSO_4$ and concentrated under reduced pressure. The residue was purified by silica gel column chromatography (petroleum ether:EtOAc = 20:1) to give 3 (1.4 g, 5.6 mmol, cis:trans = 23:77) in 83% yield. TLC: Rf 0.34 (n-hexane:EtOAc = 1:1); 1H-NMR ($CDCl_3$): δ 0.91-1.00 (m, 3H), 1.45-1.67 (m, 4H), 2.39-2.65 (m, 6H), 5.67-5.75 (cis isomer, m, 1H) and 6.21-6.32 (trans isomer, m, 1H), 6.72 (cis isomer, d, J = 11.7 Hz, 1H) and 6.94 (trans isomer, d, J = 15.9 Hz, 1H), 7.10-7.25 (m, 2H), 7.26-7.50 (m, 2H).

5-(2-hexylphenyl)pentanoic acid (4)

A mixture of 3 (1.4 g, 5.6 mmol) and Pd/C (10%, wet, 0.65 g) in MeOH (39 mL) and H_2O (3.9 mL) was stirred at room temperature overnight under H_2 atmosphere. The reaction mixture was filtered and the resulting filtrate was concentrated under reduced pressure to give 4 (1.4 g, 5.3 mmol) in 95% yield. TLC: Rf 0.52 (n-hexane:EtOAc = 1:1); 1H-NMR ($CDCl_3$): δ 0.92 (t, J = 6.9 Hz, 3H), 1.30-1.42 (m, 6H), 1.56-1.76 (m, 6H), 2.42 (t, J = 7.2 Hz, 3H), 2.58-2.68 (m, 4H), 7.13-7.16 (m, 4H).

2-methyl-2-propanyl [(1S)-1-(benzyloxy)-3-hydroxy-2-propanyl]carbamate (6)

To a solution of (2R)-3-(benzyloxy)-2-({[(2-methyl-2-propanyl)oxy]carbonyl}amino) propanoic acid (5, 5.2 g, 18 mmol) and Et_3N (2.9 mL, 21 mmol) in THF (50 mL) was added isobutyl chloroformate (2.5 mL, 19 mmol) dropwise at -15 °C. After the reaction mixture was stirred at -15 °C for 1 hour, the reaction mixture was filtered. The resulting filtrate was added to an aqueous solution of $NaBH_4$ (1.3 g, 35 mmol) in H_2O (15 mL) at 0 °C. After the reaction mixture was stirred at 0 °C for 1 hour, the reaction was quenched with H_2O. The resulting mixture was extracted with EtOAc and the organic layer was washed with 1N HCl and brine. The organic layer was dried with anhydrous Na_2SO_4 and concentrated under reduced pressure. The residue was purified by silica gel column chromatography (n-hexane:EtOAc = 3:1 to 2:3) to give 6 (5.2 g, 18 mmol) in 100% yield. TLC: Rf 0.52 (n-hexane:EtOAc = 1:1); 1H-NMR ($CDCl_3$): δ 1.44 (s, 9H), 2.56 (m, 1H),3.54-3.85 (m, 5H), 4.52 (s, 2H), 5.16 (m, 1H), 7.24-7.38 (m, 5H).

2-methyl-2-propanyl{(1R)-1-(benzyloxy)-3-[(3-oxido-1,5-dihydro-2,4,3-benzodioxa phosphepin-3-yl)oxy]-2-propanyl}carbamate (7)

To a solution of 6 (5.2 g, 18 mmol) in CH_2Cl_2 (20 mL) was added 1H-tetrazole (3.2 g, 45 mmol) and N,N-diethyl-1,5-dihydro-2,4,3-benzodioxaphosphepin-3-amine (7.2 g, 30 mmol). After the reaction mixture was stirred at room temperature for 1 hour, THF (60 mL) and 30% H_2O_2 aq. (5.6 mL) was added to the reaction mixture at 0 °C. The reaction mixture was stirred at room temperature for 1 hour and the reaction was quenched with 1N $Na_2S_2O_3$. The resulting mixture was extracted with EtOAc and the organic layer was washed with H_2O and brine. The organic layer was dried with anhydrous $MgSO_4$ and concentrated under reduced pressure. The residue was purified by silica gel column chromatography (n-hexane:EtOAc = 2:1 to 1:4) to give 7 (6.5 g, 14 mmol) in 74% yield. TLC: Rf 0.32 (n-hexane:EtOAc = 1:1); 1H-NMR ($CDCl_3$): δ 1.43 (s, 9H), 3.54 (dd, J = 9.6, 6.6 Hz, 1H), 3.64 (dd, J = 9.6, 4.2 Hz, 1H), 4.06 (m, 1H), 4.22-4.90 (m, 2H), 4.52 (s,1H), 5.00-5.25 (m, 5H), 7.24-7.40 (m, 9H).

(2R)-1-(benzyloxy)-3-[(3-oxido-1,5-dihydro-2,4,3-benzodioxaphosphepin-3-yl)oxy]-2-propanaminium chloride (8)

To a solution of 7 (6.5 g, 14 mmol) in 1,4-dioxane (20 mL), 4N HCl/1,4-dioxane solution (7 mL) was added. After the mixture was stirred for 2 hours at room temperature, the solvent was concentrated under reduced pressure. The resulting mixture was washed with MTBE and the precipitate was filtered off to give 8 (5.0 g, 13 mmol) in 90% yield. 1H-NMR (DMSO-d6): δ 3.65-3.74 (m, 3H), 4.18-4.35 (m, 2H), 4.56 (s, 2H), 5.02-5.14 (m, 2H), 5.40-5.51 (m, 2H), 7.20-7.54 (m, 9H), 8.35-8.45 (m, 3H), LC-MS (ELSD) RT = 0.864 min (95.7%), MS (ESI, Pos.) 364.2 (M – HCl + H)+.

N-{(2R)-1-(benzyloxy)-3-[(3-oxido-1,5-dihydro-2,4,3-benzodioxaphosphepin-3-yl)oxy]-2-propanyl}-5-(2-hexylphenyl)pentanamide (9)

To a mixture of 8 (173 mg, 0.43 mmol) and 4 (113 mg, 0.43 mmol) in CH_2Cl_2 (2 mL), EDC·HCl (124 mg, 0.65 mmol), HOBt (87 mg, 0.5 mmol) and Et_3N (0.12 mL, 0.86 mmol) were added. After the reaction mixture was stirred at room temperature for 4 hours, the mixture was quenched with H_2O and aqueous saturated $NaHCO_3$ followed by extraction with EtOAc. The organic layer was washed with brine, dried with anhydrous $MgSO_4$ and concentrated under reduced pressure. The residue was purified by column chromatography (n-hexane:EtOAc = 4:1 to 2:1 to 1:2 to 0:1) to give 9 (140 mg, 0.23 mmol) in 53% yield. TLC: Rf 0.54 (n-hexane:EtOAc = 1:2); 1H-NMR ($CDCl_3$): δ 0.89 (t, J = 7.0 Hz, 3H), 1.25-1.41 (m, 6H), 1.50-1.77 (m, 6H), 2.18-2.25 (m, 2H), 2.54-2.65 (m, 4H), 3.52 (dd, J = 9.4, 6.2 Hz, 1H), 3.64 (dd, J = 9.5, 3.9 Hz, 1H), 4.20-4.44 (m, 3H), 4.47-4.57 (m, 2H), 5.09-5.20 (m, 4H), 7.08-7.15 (m, 4H), 7.24-7.40 (m, 9H), LC-MS (ELSD) RT = 1.399 min (>99%), MS (ESI, Pos.) 608.4 (M + H)+

(2R)-2-{[5-(2-hexylphenyl)pentanoyl]amino}-3-hydroxypropyl dihydrogen phosphate (ONO-0740556)

A mixture of 9 (140 mg, 0.23 mmol) and ASCA-2 (20 mg) in MeOH (3 mL) was stirred at room temperature for 8 hours under H_2 atmosphere. The reaction mixture was filtered and the resulting filtrate was concentrated under reduced pressure to give ONO-0740556 (64 mg, 0.15 mmol) in 67% yield. 1H-NMR (DMSO-d6): δ 0.86 (t, J = 7.0 Hz, 3H), 1.22-1.38 (m, 6H), 1.42-1.62 (m, 6H), 2.08-2.17 (m, 2H), 2.50-2.60 (m, 4H), 3.10-3.93 (m, 5H), 7.06-7.15(m, 4H); 13C-NMR (DMSO-d6): δ 13.9, 22.0, 25.2, 28.6, 30.4, 30.7, 31.0, 31.6, 31.8, 35.1, 50.8, 59.7, 63.7, 125.6, 128.9, 139.6, 139.8, 171.9; LC-MS (ELSD) RT = 1.089 min (>99%), MS (ESI, Pos.) 416.3 (M + H)+.

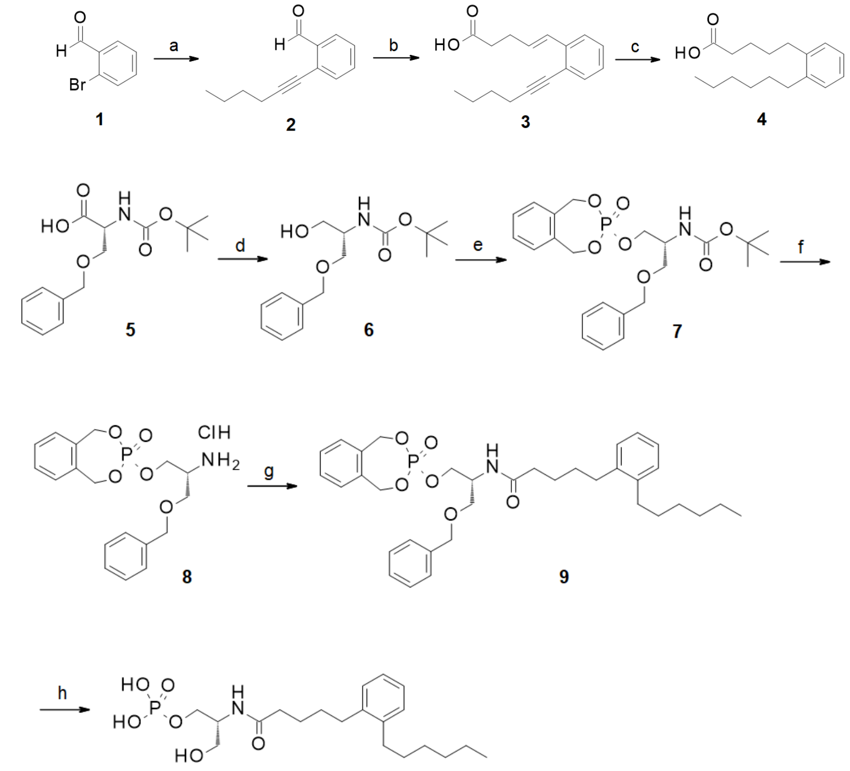

Supplementary Figure 1 | Synthesis of ONO-0740556.

Reagents and conditions: (a) 1-hexyne, Pd(dppf)Cl2, CuI, THF/Et3N, 95°C, 88%; (b) (4-carboxybutyl)(triphenyl)phosphonium bromide, NaHMDS, THF, rt, 83%; (c) H2, Pd/C, MeOH/H2O, rt, 95%.; (d) ClCO2i-Bu, Et3N, THF, -15°C; NaBH4, H2O, 0°C, quant.; (e) N,N-diethyl-1,5-dihydro-2,4,3-benzodioxaphosphepin-3-amine, 1H-tetrazole, CH2Cl2, rt; 30% H2O2 aq., THF, rt, 74%; (f) 4N HCl/1,4-dioxane, 1.4-dioxane, rt, 90%; (g) 4, EDC·HCl, HOBt, Et3N, CH2Cl2, rt, 53%; (h) H2, ASCA-2, MeOH, rt, 67%.

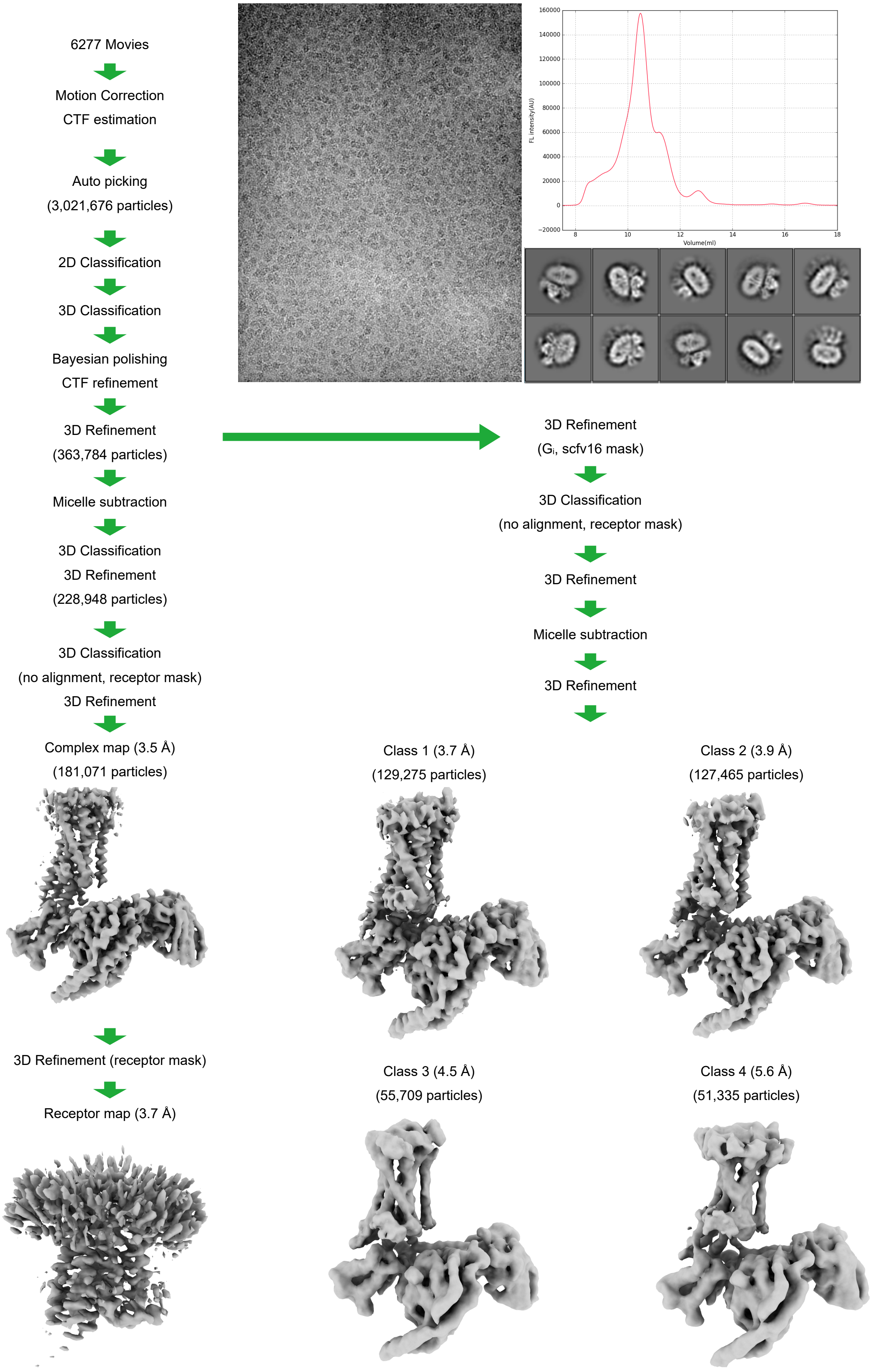

Supplementary Figure 2 | Cryo-EM analysis.
Flow chart of the cryo-EM data processing for the LPA_1-G_i complex, including particle projection selection, classification, and 3D density map reconstruction. Details are provided in the Methods section.

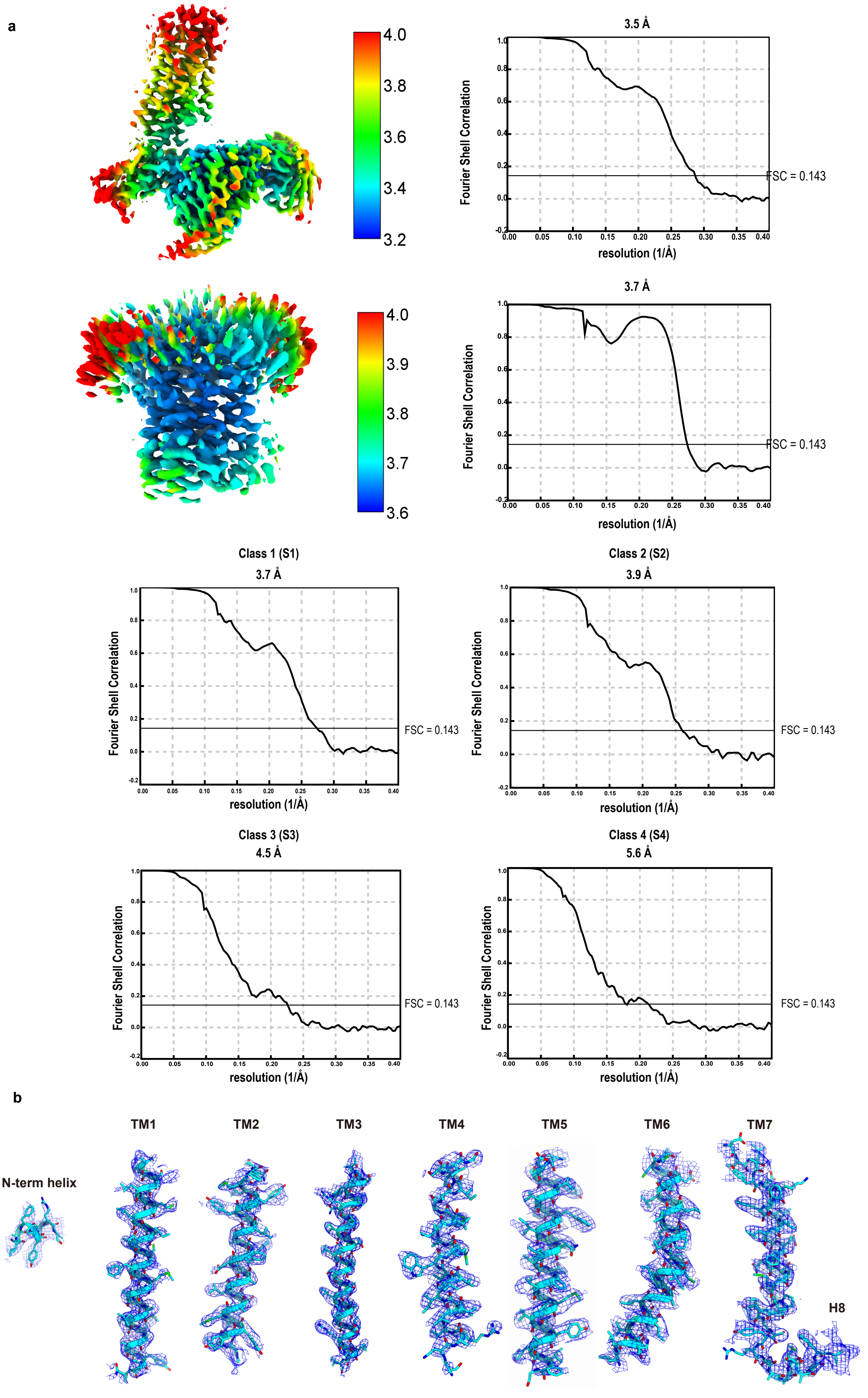

Supplementary Figure 3 | Map/model quality.

a, The local resolution maps and FSC-curves of the LPA$_1$-G$_i$ complex. **b,** The cryo-EM density map and model of the receptor are shown for all seven transmembrane α-helices, helix 8, and N-terminal helix.

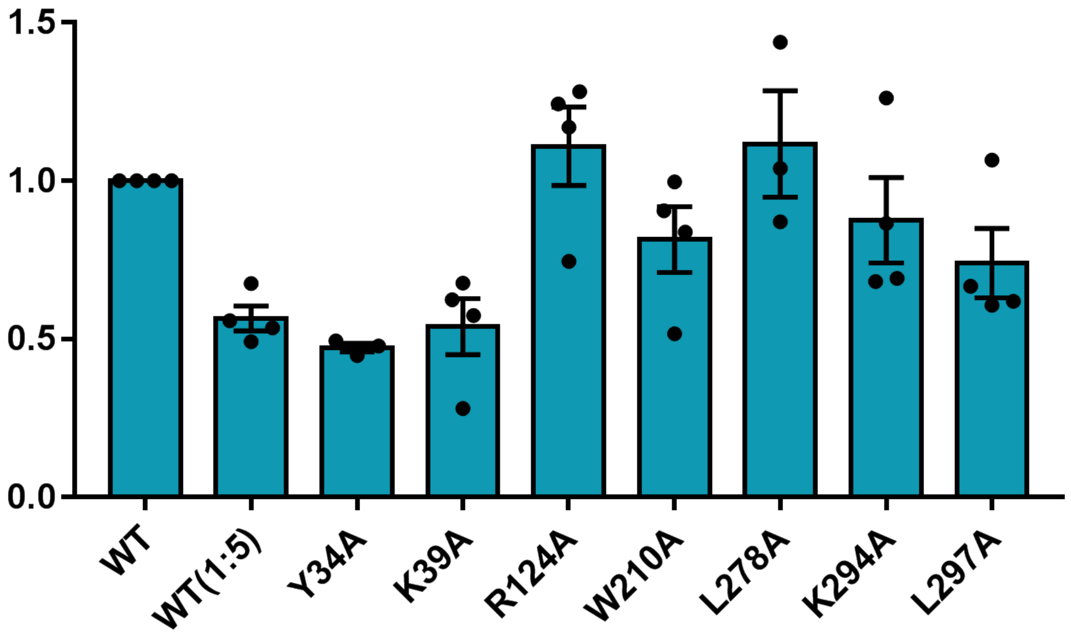

Supplementary Figure 4 | Cell surface expression levels.

Cell surface expression levels of the LPA_1-constructs. Cells transiently expressing the FLAG epitope-tagged LPA_1-constructs were labeled with an anti-FLAG HRP conjugate. Values are mean ± SEM from three or four independently performed experiments.Source data are provided as a Source Data file.

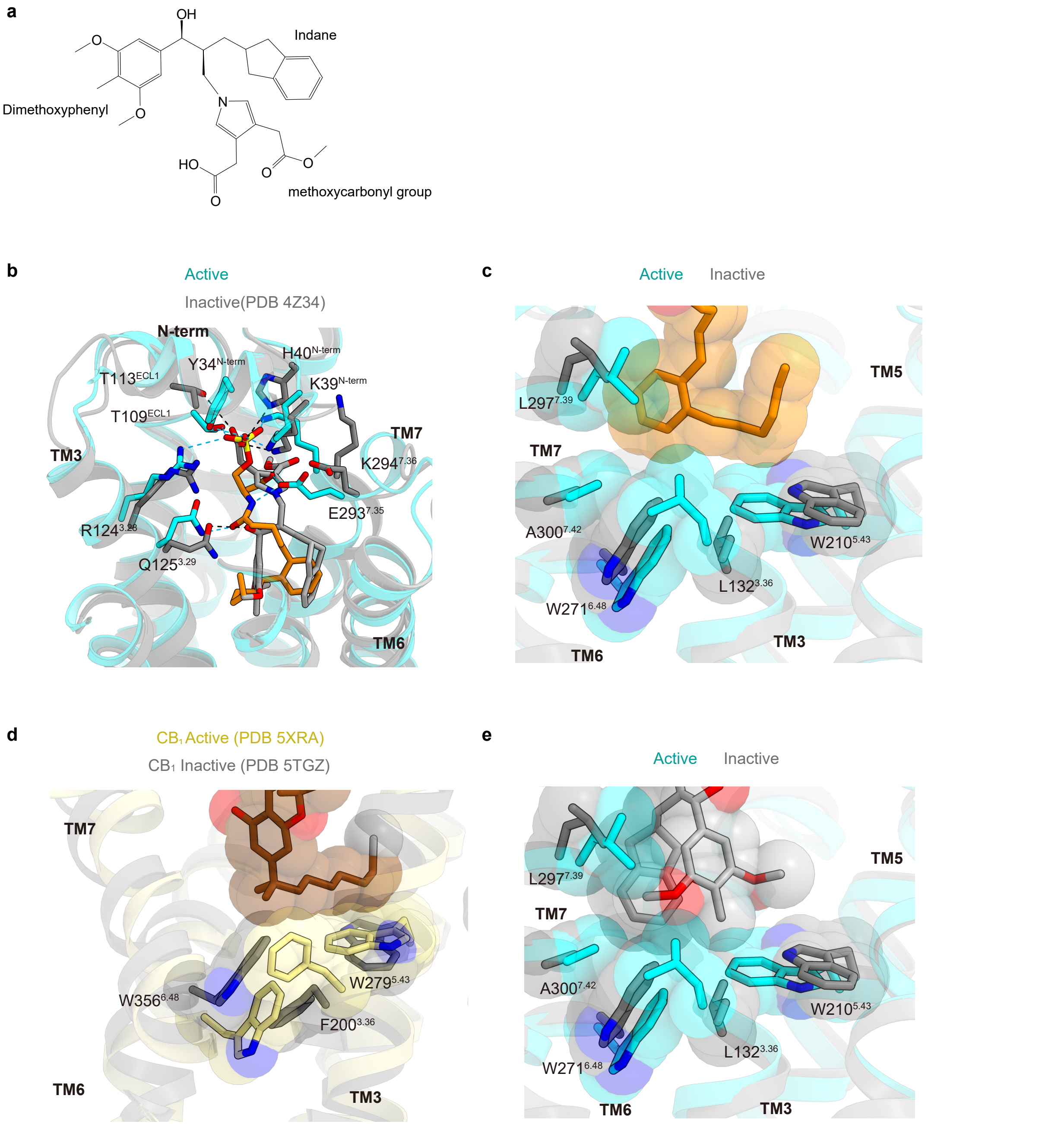

Supplementary Figure 5 | Comparison of the agonist and antagonist-bound structures.
a, Chemical structures of LPA_1 antagonist (ONO9780307). **b,** Superimposition of the antagonist and agonist-bound LPA_1 structures, colored gray (PDB 4Z34) and cyan, respectively, focused on the poler recognition region. **c, d,** Structural rearrangement of the residues constituting the bottom of the pocket upon agonist binding in the LPA_1 (c) and CB_1 (PDB 5XRA and 5TGZ) (d) structures. e, Antagonist binding mode with the bottom of the pocket in LPA_1.

.

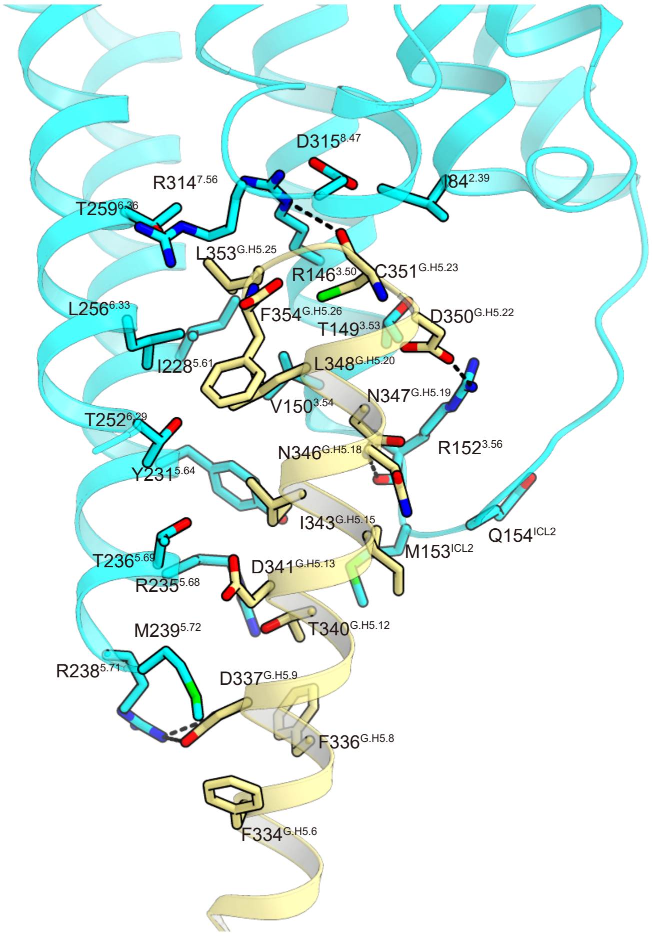

Supplementary Figure 6 | G_i interface.
Residues within 4.0 Å of the α5-helix of G_{i1} are shown as sticks.

.

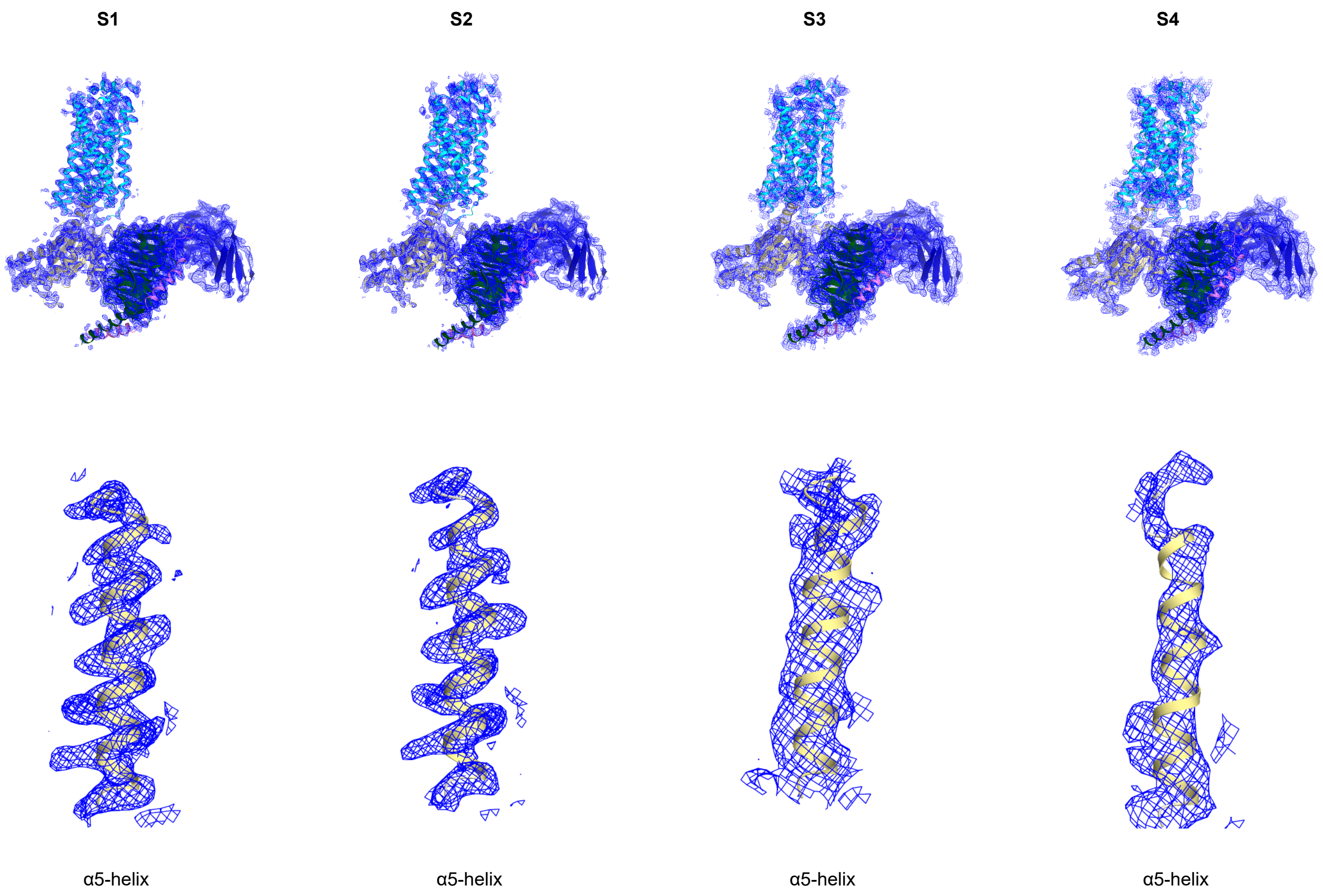

Supplementary Figure 7 | Density maps.
Density maps of the S1-4 and their α5-helices are overlaid with the ribbon models.
